# EzCatDB: the enzyme reaction database, 2015 update

**DOI:** 10.1093/nar/gku946

**Published:** 2014-10-16

**Authors:** Nozomi Nagano, Naoko Nakayama, Kazuyoshi Ikeda, Masaru Fukuie, Kiyonobu Yokota, Takuo Doi, Tsuyoshi Kato, Kentaro Tomii

**Affiliations:** 1 Computational Biology Research Center (CBRC), National Institute of Advanced Industrial Science and Technology (AIST), Tokyo Waterfront Bio-IT Research Building, 2-4-7 Aomi, Koto-ku, Tokyo 135-0064, Japan; 2 Level Five Co. Ltd., Grove Tower 4112, 4-21-1, Shibaura, Minato-ku, Tokyo 108-0023, Japan; 3 Faculty of Science and Engineering, Gunma University, 1-5-1 Tenjin-cho, Kiryu, Gunma 376-8515, Japan; 4 Center for Informational Biology, Ochanomizu University, 2-1-1 Otsuka, Bunkyo-ku, Tokyo 112-8610, Japan

## Abstract

The EzCatDB database (http://ezcatdb.cbrc.jp/EzCatDB/) has emphasized manual classification of enzyme reactions from the viewpoints of enzyme active-site structures and their catalytic mechanisms based on literature information, amino acid sequences of enzymes (UniProtKB) and the corresponding tertiary structures from the Protein Data Bank (PDB). Reaction types such as hydrolysis, transfer, addition, elimination, isomerization, hydride transfer and electron transfer have been included in the reaction classification, RLCP. This database includes information related to ligand molecules on the enzyme structures in the PDB data, classified in terms of cofactors, substrates, products and intermediates, which are also necessary to elucidate the catalytic mechanisms. Recently, the database system was updated. The 3D structures of active sites for each PDB entry can be viewed using Jmol or Rasmol software. Moreover, sequence search systems of two types were developed for the EzCatDB database: EzCat-BLAST and EzCat-FORTE. EzCat-BLAST is suitable for quick searches, adopting the BLAST algorithm, whereas EzCat-FORTE is more suitable for detecting remote homologues, adopting the algorithm for FORTE protein structure prediction software. Another system, EzMetAct, is also available to searching for major active-site structures in EzCatDB, for which PDB-formatted queries can be searched.

## INTRODUCTION

Systematic classifications of enzyme reactions have been crucially important to ascertain the relations between enzyme structures and functions, although they are extremely time-consuming. The Enzyme Commission (E.C.) (or NC-IUBMB; http://www.chem.qmul.ac.uk/iubmb/enzyme/) classifies enzymes hierarchically by manually assigning a four-digit number to each enzyme based on the chemical structures of substrates and products involved in catalysis (1–3). This classification has been widely used for a long time. However, the E.C. classification does not consider enzyme sequences, structures and mechanisms, which are extremely important for elucidating the enzymatic reactions. The EzCatDB database (4) has emphasized manual classification of enzyme reactions in terms of enzyme active-site structures and their catalytic mechanisms based on information from the literature, amino acid sequences of enzymes (UniProtKB) (5) and the corresponding tertiary structures from the Protein Data Bank (PDB) (6). In this database, the reaction classification, Reaction, Ligand, Catalysis and Protein active-site (RLCP), which reflects the types of catalytic mechanism and enzyme active-site structures as well as basic reaction types and ligand types, has been developed since the first release of this database in 2005 (4). Although the RLCP classification originally included only the reaction types such as hydrolysis and transfer reactions at the first database release, it has now been extended to include other reaction types such as addition, elimination, isomerization, hydride transfer and electron transfer reactions. As for other major enzyme reaction databases, the Catalytic Site Atlas (CSA) (7) annotates enzyme functional residues in the enzyme entries of PDB (6), whereas the MACiE database (8) annotates and classifies each reaction step in detail, rather than the overall reaction, which usually consists of several reaction steps. Consequently, the EzCatDB database plays a distinct role in enzyme research from these other enzyme reaction databases because it classifies overall reactions.

Moreover, the EzCatDB database includes information related to ligand molecules on the enzyme structures in the PDB data, classified in terms of cofactors, substrates, products and intermediates, which are also necessary to elucidate the catalytic mechanisms. Particularly, it has provided information related to intermediates, which must be extremely important for catalytic reactions. Regarding the database entries, although they were only 362 in the first release in January of 2005, they have been increased to 871 in the current version. More recently, the database system was updated. Recent developments include novel search systems, as described below. The updated EzCatDB is available at http://ezcatdb.cbrc.jp/EzCatDB/.

## BASIC REACTIONS IN RLCP CLASSIFICATION

As described earlier in the literature (4), the reaction classification in EzCatDB clusters catalytic reactions at four levels: basic reactions (R), ligand groups (L), types of catalytic mechanism (C) and active sites located on enzyme proteins (P). The ‘basic reaction’ group includes reaction types, which can be related to the primary number of E.C. numbers (4). At the first release of the EzCatDB database, 10 years ago, this classification included only hydrolysis, phosphorolysis and transfer reactions (4). Since then, the RLCP classification has been extended to include the following other reaction types:

- Addition reaction (additive double-bond deformation): A reaction that adds a group to a double bond, in which the double bond is converted to a single bond. This reaction is an inverse reaction of ‘elimination reaction’ described below.
- Elimination reaction (eliminative double-bond formation): A reaction that eliminates a group from a moiety that consists of a single bond, in which the single bond can be converted to a double bond. This reaction is an inverse reaction of ‘addition reaction’ described above.
- Exchange of double-bonded atoms: A reaction that replaces double-bonded atoms. This reaction usually involves a Schiff-base bond, in which a carbonyl group is replaced by a Schiff-base bond, and vice versa; alternatively, an external/internal aldimine group is converted on the active sites of pyridoxal 5′-phosphate-dependent enzymes.
- Shift of the double-bond position (or isomerization): A reaction that shifts the position of a double bond to its adjacent bond.
- Hydride transfer: A major redox reaction that catalyzes transfer of one proton and two electrons between substrate/intermediate and cofactor such as NAD(H) and NADP(H). This reaction is distinct from the electron transfer described below.
- Electron transfer: A major redox reaction that catalyzes transfer of an electron, usually between heavy metals or sulfur atoms that are involved in redox reactions.

Reaction types such as addition (a), elimination (b), exchange of double-bonded atoms (c) and isomerization (d) involve double bonds, in contrast to hydrolysis and transfer reactions that involve nucleophilic substitutions. Addition reaction (a) adds a group to a double bond, whereas elimination reaction (b) removes a group from a single bond, leading to formation of a new double bond. Reaction (c) replaces double-bonded atoms (examples: C=O to C=N and vice versa). Isomerization in our definition (d) catalyzes shifts of the position of double bonds. For example, triose phosphate isomerase (EzCatDB ID; S00225) catalyzes two consecutive isomerization reactions, in which a shift of the double-bond position occurs twice. In the case of mandelate racemase (EzCatDB ID; D00273), it also catalyzes two consecutive isomerization reactions. During the two reactions, a proton is removed from one side, forming an enolic intermediate, in the first isomerization, and another proton is attached from the opposite side in the second isomerization. Consequently, these two isomerization reactions complete the racemization. On the other hand, reaction types such as hydride transfer (e) and electron transfer (f) are major redox reactions. In the future, other redox reactions such as oxidation can be included in the RLCP reaction classification. Although the original RLCP site page (URL: http://ezcatdb.cbrc.jp/EzCatDB/RLCP/index.html) is hierarchic, the table of the whole reaction classification can be viewed at the RLCP overview site (URL: http://ezcatdb.cbrc.jp/EzCatDB/overview/reaction.html).

## LIGAND DATA IN EzCatDB

For each entry of EzCatDB, annotation of ligand molecules bound to the enzyme structures in the corresponding PDB entries (6) was performed manually and was tabulated for corresponding substrates, products and cofactors (4). Because the compound data for substrates, products and cofactors in EzCatDB are based on KEGG compound data (9), most of the compound data in this database are assigned KEGG compound IDs, which have six-digit codes with an initial C (such as C00010 for Coenzyme A). However, some compounds are not assigned by any particular compound IDs. For instance, tryptophan tryptophylquinone is an essential cofactor for ethylamine dehydrogenase (EC 1.4.9.1), but this compound is not assigned any KEGG compound ID. In such a case, a corresponding compound datum was created in EzCatDB, based on literature information, and a ligand ID, which has a six-digit code with an initial L (such as L00002 for tryptophan tryptophylquinone), was assigned. In the case of intermediates, corresponding compound data are usually not registered in other enzyme databases, although intermediates are necessary in enzyme reactions, and their corresponding 3D-structural data can be included in enzyme structural data in PDB (6). Therefore, intermediate compound data are also created, based on literature information, and intermediate IDs, which have six-digit codes with an initial I (such as I00001 for enolpyruvate), are assigned to the intermediate data. For compound data that are created for EzCatDB, mol-formatted data are available, in addition to the JPEG-formatted image data for chemical structures, at each compound site in EzCatDB.

## RECENT DEVELOPMENTS

Since the first release of this database, various improvements have been made in EzCatDB (4). The main features of the updated systems are described in the following sections.

### Table of annotated ligand/active-site data for the PDB entries

The 3D structures of active sites for each PDB data can be viewed using Jmol (10) or Rasmol (11) software, based on manually annotated active-site residues and ligand data from PDB (6). In addition to the PDBsum link (12), a link to another PDB related database, PDBj (13), is also available. Moreover, mmCIF-formatted active-site structure data are downloadable because this format (14) is a common and widely used alternative to the PDB format.

### Three novel search systems: EzCat-BLAST, EzCat-FORTE and EzMetAct

Sequence search systems of two types, EzCat-BLAST and EzCat-FORTE, were developed for different uses for the EzCatDB database. EzCat-BLAST is suitable for quick searches, adopting the BLAST algorithm (15,16), whereas EzCat-FORTE is more suitable for the detection of remote homologues, adopting the algorithm for FORTE protein structure prediction software (17,18).

**Figure 1.**
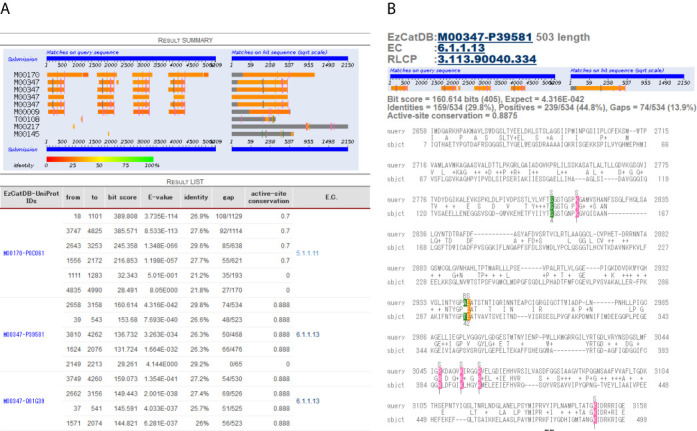
Output for an NRPS sequence (UniProtKB; B8NXQ7) by EzCat-BLAST. (**A**) Top page. In the ‘RESULT SUMMARY’, sequence matches between query and hit sequences are shown as colored bars along with active-site matches. For this query sequence, four adenylation domains of NRPS, which are homologous to the representative sequences from EzCatDB entries, M00170, M00347 and M00009, were detected. In the ‘RESULT LIST’, the corresponding EzCatDB and UniProtKB IDs for the top hit sequences are shown with the position of the query sequence, bit score, E-value and active-site conservation for each hit sequence. (**B**) Alignment display for the query and hit sequences. Active-site residues are colored according to the type of active-site residues. Sidechain catalytic residues are presented in magenta, whereas mainchain catalytic residues are shown in green. The cofactor binding residue is shown in orange. The link to EzCatDB entry is also shown with the alignment data.

For the EzCat-BLAST system, sequence data of the UniProtKB and corresponding PDB data registered in the EzCatDB, which are assigned with manually annotated active-site residue information, are clustered using BLASTClust software (with 70% sequence identity threshold and 3% coverage), which is also a part of the BLAST package (15,16), so that each UniProtKB sequence can be clustered together with the corresponding PDB sequence data that have annotated active-site information. Although most EzCatDB entries only include a single UniProtKB sequence, some entries include more than one sequence. In such a case, close homologues can be removed by the clustering process. The active-site residue information can be mapped onto the representative sequence data from the PDB sequence data, which are clustered together, using multiple-alignment software, POA version 2.0 (19,20), along with the substitution matrix BLOSUM62 (21). Therefore, the dataset of representative sequences that have active-site residue information can be prepared. BLASTP search (15,16) can be performed against the database that consists of the representative sequence dataset in the EzCat-BLAST. A display system for EzCat-BLAST was also developed, so that the active-site residue matches of the query sequence with the hit sequences can be viewed (Figure 1). In the EzCat-BLAST system, the active-site conservation was defined as follows. If the hit sequence includes any corresponding active-site residues, then the active-site conservation must be calculated as 0–1. If the figure is one, then the query sequence is presumed to have all the active-site residues, which are completely conserved in the corresponding representative sequence. According to the chemical similarities between the query residue and the hit active-site residue, the weight was assigned to the matched residue. If the matched residue is the same or chemically similar (such as Asp and Glu), then it would be assigned a higher weight. Consequently, by summing up the weighted matched residues, the score for the hit active-site residues could be obtained. To calculate the active-site conservation, the score is divided by the total number of active-site residues, which are on the hit representative sequence. If the hit representative sequence contains no active-site residue, then the active-site conservation will not be calculated. This system is suitable for quick searches, and also for sequences longer than 800 residues that might consist of multiple domains, since the BLAST algorithm adopts local alignment (15,16). For instance, the sequence data for large enzymes such as polyketide synthases (PKS) and non-ribosomal peptide synthetases (NRPS) tend to be larger than 800 residues, with multiple domains. It has been confirmed that sequences with up to 7500 residues can be sought using this EzCat-BLAST system. In Figure 1, the output page of the sequence of NRPS (UniProtKB; B8NXQ7) by EzCat-BLAST is shown. For NRPS sequences, representative sequence data that contain adenylation domain (EzCatDB IDs; M00170, M00347 and M00009) can be hit. In some cases, sequences that contain acyl carrier protein (EzCatDB IDs; M00347 and U00001) can be hit, although this domain is extremely difficult to detect. In Figure 1, four adenylation domains were detected. In contrast, β-ketoacyl transferase (EzCatDB IDs; D00871, D00867, D00825 and D00826), acyltransferase (EzCatDB ID; D00091) and β-ketoacyl reductase (EzCatDB IDs; S00328 and S00553) can be detected for PKS sequences. Either acyl carrier protein (EzCatDB IDs; M00347 and U00001) or thioesterase (EzCatDB ID: S00919) can also be hit for PKS, although these domains are extremely difficult to detect.

**Figure 2.**
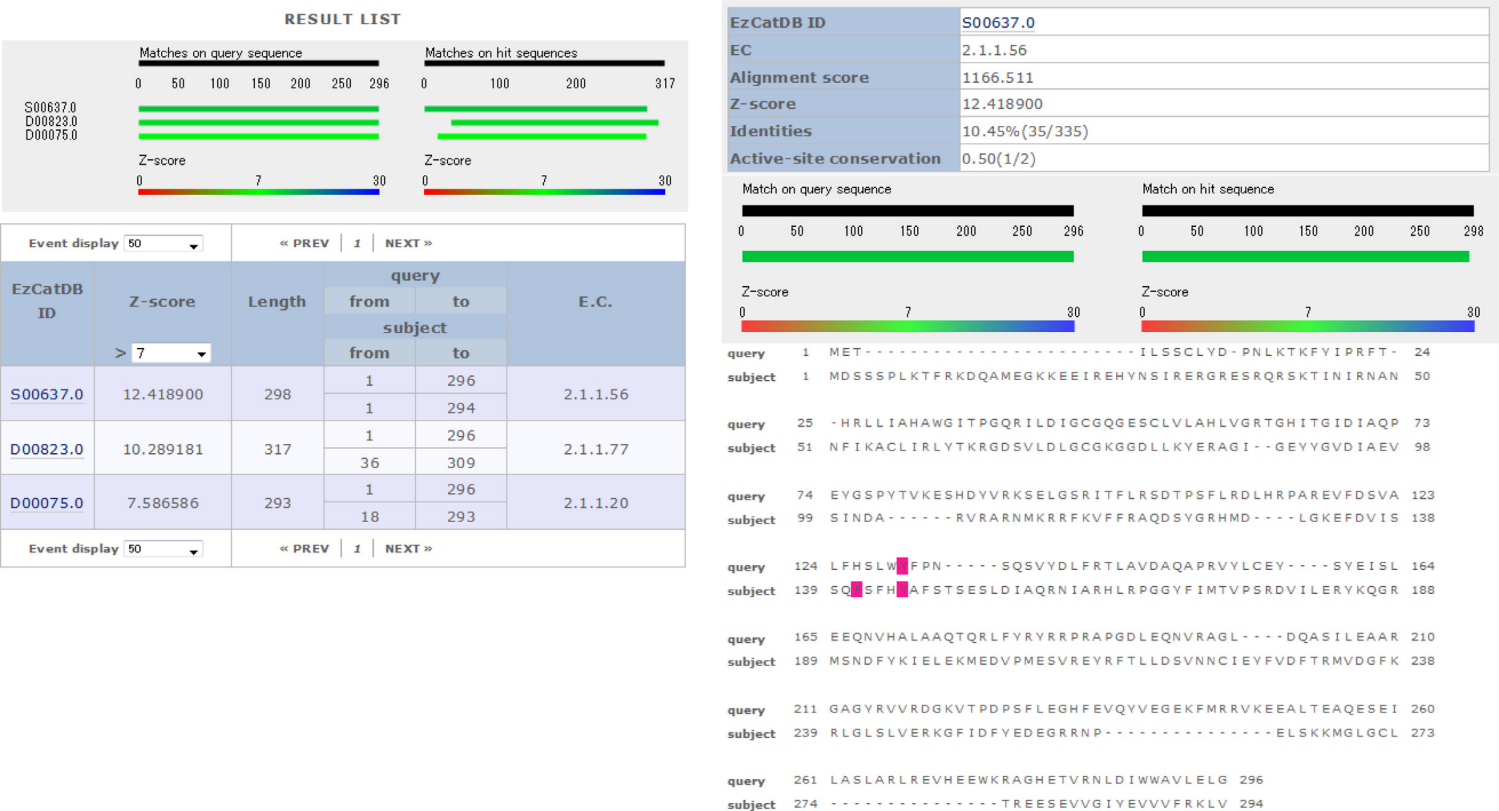
Output for a methyltransferase sequence (UniProtKB; Q2U5R7) by EzCat-FORTE. In the ‘RESULT LIST’ of top page, sequence matches between query and hit sequences are shown by colored bars. The corresponding EzCatDB IDs for the hit sequences are presented with Z-score, positions of query and hit sequences and E.C. numbers for each hit. For this query sequence, a domain for methyltransferase, which is homologous to the representative sequences from EzCatDB entries, S00637, D00823 and D00075, can be detected. In the alignment display, alignment of the query and top hit sequence (EzCatDB ID; S00637) are shown. Active-site residues for the hit sequence are colored. If the matched residue of the query sequence is the same as the active-site residues of the hit sequence, then it can also be colored. The link to EzCatDB entry is also shown in the table of the alignment display.

In the EzCat-FORTE system, the position-specific scoring matrix (PSSM) is pre-calculated for each representative sequence that contains active-site information, according to the method for FORTE that was developed originally for protein structure prediction (17,18). When a user provides a query sequence to this system, the PSSM is also created for the query sequence, enabling comparison with the PSSMs prepared for the EzCatDB representative sequences. Consequently, even sequences of remote homologues can be searched against EzCatDB to find corresponding sequences. Moreover, in contrast to EzCat-BLAST, which adopts local alignment, this FORTE system adopts the global–local algorithm (17,18). Even if no active-site residue is detectable with EzCat-BLAST, EzCat-FORTE can be useful to detect active-site residue positions. Figure 2 shows the EzCat-FORTE output page of the sequence of a methyltransferase (UniProtKB; Q2U5R7). For data of this sequence, methyltransferase sequences can be only partially detected using EzCat-BLAST, without any significant score or any matched position with active-site residues. However, EzCat-FORTE was able to identify the remote homologous methyltransferase (EzCatDB IDs: S00637, D00823 and D00075) (Figure 2). For the top hit sequence, the active-site positions were identified. One of the matched residues was conserved in the query sequence (Figure 2). In the case of large proteins, the sequence region of the large protein, in which no enzymatic domain could be identified using EzCat-BLAST, can be analyzed using EzCat-FORTE. Therefore, a sequence region that one might want to analyze further in greater detail can be identified using the EzCat-FORTE program, after pre-scanning for large proteins using the EzCat-BLAST program.

Currently, 1413 representative sequences from 871 EzCatDB entries are available for EzCat-BLAST and EzCat-FORTE. These representative sequences can be prepared semi-automatically.

Another system, EzMetAct, is also available to search for major active-site structures in EzCatDB, for which PDB-formatted queries can be searched (22). Analysis of the enzyme reactions from EzCatDB revealed that several analogous reactions can be observed in non-homologous enzymes (23). For example, active sites of serine proteases, such as trypsin and subtilisin, tend to share similar local structures, even if they are not mutually homologous (24). In such cases, EzMetAct is expected to be useful to detect similar active-site structures (22). This EzMetAct system adopts a template-based method, in which the weights for the atoms in the template are determined using a metric learning algorithm, a kind of machine-learning algorithm (22). For example, the structure of immunoglobulin A1 protease (PDB; 3h09: peptidase S6 family) can be analyzed using EzMetAct, predicting that His100, Asp164, Gly286 and Ser288 are identified as active-site residues for the serine protease, although the two sequence search systems cannot detect its active-site residues because of a lack of homologous enzyme deposited in the EzCatDB database. Currently, 192 templates from 103 EzCatDB entries are available for this system because the template preparation has not been automated yet.

Taken together, these three systems are expected to be extremely helpful and convenient for searching for EzCatDB entries.

## CONCLUSION

The EzCatDB enzyme reaction database was updated substantially. The recent developments include three novel search systems, two sequence search systems, EzCat-BLAST and EzCat-FORTE, and an active-site structure search system, EzMetAct. Using these systems, the database is expected to become more useful and convenient for users. In addition to other databases such as UniProtKB (5), PDBsum (12), PDBj (13), CATH (25) and KEGG (9), EzCatDB makes links to other major enzyme reaction databases such as CSA (7) and MACiE (8) if corresponding enzyme entries exist. Although it has not made links to other enzyme databases, such as Structure-Function Linkage Database (26), the carbohydrate-active enzymes database (27) and the α/β-hydrolase fold superfamily database (ESTHER) (28), we are planning to make links from EzCatDB to those enzyme-related databases in the near future. Currently, the database contains 871 enzyme entries, related to 1610 UniProtKB sequences and 6704 PDB entries (Table 1). Because many enzymes are to be included in this database, we are still preparing 300 more EzCatDB entries, which will be registered in the future. Downloadable files for the entire dataset are available at http://ezcatdb.cbrc.jp/EzCatDB/DL/index.html. The general help page for this database is also available at http://ezcatdb.cbrc.jp/EzCatDB/ezcat.html.

**Table 1.** Current contents of EzCatDB

Related data	Description^a^	Number of corresponding data^a,b^
DB codes for EzCatDB	Entry ID in this EzCatDB database	871 EzCatDB entries
RLCP classification	Hierarchic classification of reaction mechanisms	516 EzCatDB entries for 439 RLCP classes
Compound data	Compound data for substrates/products/cofactors	1106 compound data
		1020 KEGG compounds (9) /86 EzCatDB compounds
Intermediate data	Compound data for intermediates	151 intermediate data
		3 KEGG compounds (9) /148 EzCatDB intermediates
E.C. numbers	Enzyme commission number (1–3)	667 E.C. numbers
Catalytic domains	Catalytic domains of enzyme structures that have active-site residues, based on CATH classification (25).	335 CATH domains (25)
UniProtKB	Protein sequence data from UniProtKB (5)	1612 sequences (1413 representative sequences)
PDB	Tertiary structure data from Protein Data Bank (6)	6704 PDB entries (6) for 871 EzCatDB entries

^a^Reference numbers are shown in parentheses.

^b^The number of data might change through update.
